# A therapeutic cancer vaccine against GL261 murine glioma

**DOI:** 10.1186/s12967-015-0757-9

**Published:** 2016-01-05

**Authors:** Mark S. Kindy, Jin Yu, Hong Zhu, Michael T. Smith, Sebastiano Gattoni-Celli

**Affiliations:** Department of Pharmaceutical Sciences, University of South Florida, Tampa, FL 33612 USA; Department of Pathology & Laboratory Medicine, Medical University of South Carolina, Charleston, SC 29425 USA; Department of Radiation Oncology, Medical University of South Carolina, Charleston, SC 29425 USA; Ralph H. Johnson VA Medical Center, Charleston, SC 29401 USA

**Keywords:** Glioblastoma, Immunotherapy, Semi-allogeneic, Cancer, Vaccine

## Abstract

**Background:**

Glioblastoma (GBM) is the deadliest of brain tumors. Standard treatment for GBM is surgery, followed by combined radiation therapy and chemotherapy. Current therapy prolongs survival but does not offer a cure. We report on a novel immunotherapy against GBM, tested in an animal model of C57BL/6 mice injected intra-cranially with a lethal dose of murine GL261 glioma cells.

**Methods:**

Ten week-old C57BL/6 mice were anesthetized before injection of 2 × 10^4^ GL261 cells in the right cerebral hemisphere and after 3 days half of the mice were administered a single subcutaneous (s.c.) injection of irradiated semi-allogeneic vaccine, while mock-vaccinated mice received a s.c. injection of phosphate-buffered saline (PBS). Tumor engraftment was monitored through bioluminescence imaging (BLI). Length of animal survival was measured by Kaplan–Meier graphs and statistics. At time of sacrifice brain tissue was processed for estimation of tumor size and immunohistochemical studies.

**Results:**

Overall survival of vaccinated mice was significantly longer compared to mock-vaccinated mice. Five to ten percent of vaccinated mice survived more than 90 days following the engraftment of GL261 cells in the brain and appeared to be free of disease by BLI. Tumor volume in the brain of vaccinated mice was on average five to ten-fold smaller compared to mock-vaccinated mice. In vaccinated mice, conspicuous microglia infiltrates were observed in tumor tissue sections and activated microglia appeared to form a fence along the perimeter of the tumor cells. The results of these animal studies persuaded the Office of Orphan Products Development of the Food and Drug Administration (FDA) to grant Orphan Drug Designation for treatment of GBM with irradiated, semi-allogeneic vaccines.

**Conclusions:**

Our preclinical observations suggest that semi-allogeneic vaccines could be tested clinically on subjects with GBM, as an adjuvant to standard treatment.

## Background

Glioblastoma multiforme (GBM) is the deadliest of brain tumors and is one of a group of tumors referred to as gliomas. GBMs make up approximately 15 % of all primary brain tumors [1]. Classified as a Grade IV (most serious) astrocytoma, GBMs develop from the astrocytes that support nerve cells, primarily in the cerebral hemispheres, but can develop in other parts of the brain, brainstem, or spinal cord. Each year, more than 3000 Americans are diagnosed with GBMs. The cure rate for GBMs is grim, with current therapy prolonging survival but not offering a cure.

Standard treatment for GBM is surgery, followed by combined radiation therapy and chemotherapy. GBM’s capacity to invade and infiltrate normal surrounding brain tissue makes complete resection virtually impossible. After surgery, combined chemo-radiation is used to kill residual tumor cells and to delay recurrence. Temozolomide was approved by the Food and Drug Administration (FDA) in 2005 for treatment of adult GBM; subsequently, the FDA approved Avastin (Bevacizumab) for treating GBM. Nevertheless, with standard of care therapy, the median survival of children and adults with GBM is 15 months, and the 5-year survival rate is approximately 10 % [2]. This tumor ultimately takes the life of nearly every affected patient.

It has been recently reported that VA patients with GBM showed a decreased median survival (6.5 months) relative to a national cohort of adults (9.0 months); furthermore, 1, 2, and 5 years survival rates in Veterans were 26.8, 5.4, and 0.5 %, respectively, versus 37.8, 12.8, and 4.1 %, respectively, in a comparable national cohort of adults. These GBM survival data highlight a potential disparity and a much worse clinical outcome among affected Veterans [3]; therefore, it is important to explore additional therapeutic options for treating GBM, especially in Veterans.

The capacity of T cells to recognize allogeneic major histocompatibility complex (MHC) molecules as intact structures on the surface of foreign cells is called direct T cell allorecognition and is responsible for the powerful immune reactions associated with transplant rejection, a phenomenon called “alloagression” [4]. To a large extent this is due to the ability of allogeneic stimulation to mobilize up to 10 % of all T lymphocytes, compared with a precursor T-cell frequency of between 10^−4^ and 10^−5^ for most common antigens. At the same time, each of the lymphocytes activated through direct allorecognition will also recognize specific antigenic peptides presented in the context of a self-MHC molecule (MHC restriction). Cross-reactivity between alloantigens and self MHC-restricted antigens can be harnessed to target tumor-associated antigens [4].

Experimental results from studies with inbred mice and their syngeneic tumors have indicated that the inoculation of semi-allogeneic cell hybrids (derived from the fusion between syngeneic tumor cells and an allogeneic cell line) protects the animal host from a subsequent lethal inoculation with unmodified syngeneic tumor cells [5, 6]. Semi-allogeneic somatic cell hybrids were generated by fusing EL-4 T-lymphoma cells (H-2^b^) and BALB/c-derived renal adenocarcinoma RAG cells (H-2^d^); these hybrids were injected intra-peritoneally (i.p.) in C57BL/6 mice (H-2^b^). Vaccination with irradiated allogeneic cells alone or syngeneic tumor cells alone did not provide significant protection against a tumorigenic challenge with EL-4 cells [5]. The results of these studies also showed that the enhanced immunity was not due simply to an allogeneic effect. In fact, co-administration (injection) into experimental mice of allogeneic cells together with irradiated autologous tumor cells (i.e., without fusion) did not protect them from a subsequent inoculation with autologous tumor cells, supporting the conclusion that, in order to achieve maximum anti-tumor protection, the tumor-associated antigen and the alloantigen needed to be on the same cell [5].

Irradiated semi-allogeneic tumor cell hybrids conferred protection against a subsequent tumorigenic inoculation of EL-4 cells; in contrast, control mice that were mock-vaccinated with i.p.-injected phosphate-buffered saline (PBS) were killed by EL-4-derived lymphomas, which grew rapidly to a large size in the peritoneal cavity [6]. Focused microarray analyses performed on RNA purified from splenocytes of vaccinated (protected) mice revealed that expression of interferon (INF)-γ was upregulated while programmed cell death protein 1 (PD-1) expression was down-regulated compared to splenocyte RNA from control mice, suggesting that semi-allogeneic vaccines are able to activate cytotoxic T cells and interfere with, or even block, the tumor-mediated induction of immune tolerance, a key mechanism underlying the suppression of anti-tumor immunity in the immune competent host [6].

In an attempt to extend this immunotherapy approach to brain tumors, we tested semi-allogeneic vaccines in a mouse model of GBM. The mouse glioma 261 (GL261) was originally established by intracranial injection of 3-methylcholantrene into C57BL/6 mice (H-2^b^ haplotype), and serially passaged intracranially or subcutaneously as tissue tumor pieces in syngeneic mice [7]. Subsequently, GL261-derived cell cultures were established to perform biological and immunological studies with the objective of investigating new treatment modalities for GBM [8]. This research team also reported that subcutaneous (s.c.) vaccination with irradiated GL261 cells was partially effective in a preventing the engraftment of GL261-derived brain tumors, but totally ineffective in a therapeutic setting, i.e., when administered on the day of intracranial tumor inoculation or 3 days thereafter [8].

## Methods

### Cell lines

GL261 cells were a generous gift from Dr. Michael R. Olin (University of Minnesota), and were tested and found free of adventitious agents, before being injected into mice. GL261 cells (H-2^b^ haplotype) were cultured in Dulbecco’s modified Eagle’s medium (DMEM, Invitrogen), added with 10 % of fetal bovine serum (FBS, Atlanta Biologicals). We also tested the resistance of these cells in medium containing various concentrations of the neomycin analog Geneticin (G418), and found that a medium containing 200 µg/mL of G418 could kill GL261 within 2 weeks. Our stock of GL 261 cells also expresses a transfected firefly luciferase gene, which allow us to monitor tumor engraftment by bioluminescence imaging technology (see below). RAG cells were purchased from the American Type Culture Collection (ATCC), tested and found free of adventitious agents, before being injected into mice. RAG cells were cultured in DMEM added with 10 % FBS. RAG cells are deficient in the X-linked hypoxanthine-guanine phosphoribosyl transferase gene (HGPRT^−^). They are a non-reverting, 8-azaguanine-resistant clone of the Renal-2a cell line, originally derived from a kidney adenocarcinoma of BALB/c origin (H-2^d^ haplotype); therefore, they are killed in culture media containing a supplement of hypoxanthine, aminopterin, and thymidine (HAT, Invitrogen). We have previously confirmed that RAG cells are non-reverting, 8-azaguanine-resistant, and HAT-sensitive [5, 6]. Neomycin-resistant RAG cells were generated by DNA-mediated gene transfer of the pRSV-neo plasmid (RAG-neo), as previously described [9]. RAG-neo cells can grow in medium containing >400 µg/mL of G418. To generate semi-allogeneic somatic cell hybrids, single-cell suspensions of GL261 and RAG-neo cells were mixed at a 3:1 ratio in serum-free DMEM containing 50 μM sodium dodecyl sulfate (SDS) and centrifuged at 300×*g* for 5 min (min) at room temperature. The mixed cell pellet was then slowly suspended in 1 mL 50 % polyethylene glycol (PEG)-1450 (cell-culture grade from the ATCC and diluted in serum-free DMEM) over a 1 min period while gently stirring. The cell suspension was then slowly diluted over a 2 min period with DMEM supplemented with 10 % FBS. After centrifuging at 300xg for 5 min, fused cells were plated in selective medium (DMEM + 10 % FBS, containing 400 µg/mL G418 and HAT supplement). Under these culture conditions only RAG x GL261 semi-allogeneic somatic cell hybrids can survive, since RAG-neo cells are killed by aminopterin and GL261 cells are killed by G418.

### Fluorescence-activated cell sorting (FACS)

Drug-resistant cultures were expanded and tested by FACS for co-expression of MHC surface antigens using phycoerythrin (PE)-labeled, H-2K^b^-specific monoclonal antibody (MAb) AF6-88.5 (BD Bioscience cat.# 553570), and allophycocyanin (APC)-labeled, H-2K^d^-specific MAb SF1.1 (eBioscience cat.# 17-5957-82).

### Animals

Mouse studies were reviewed and approved by the Institutional Animal Care and Use Committee (IACUC) and were conducted in accordance with all applicable national and institutional guidelines for the care and use of animals. Ten to twelve week-old C57BL/6 male mice (Jackson Laboratories) were anesthetized by i.p. injection of Ketamine (JHP Pharmaceuticals—90 mg/per kg) and Xylazine (Lloyd Laboratories—10 mg/kg), and positioned in a Kopf stereotaxic frame for intracranial injection into the *striatum* (2.2 mm medio-lateral, 0.2 mm anterior-posterior, and 3 mm dorso-ventral). Injection into the right cerebral hemisphere of GL261 cells suspended in PBS (2 × 10^4^/mouse) was performed using a 5 μL Hamilton micro-syringe under mechanical control to avoid brain injuries during the procedure. Three days after tumor inoculation, treated mice were injected s.c. with lethally irradiated [30 Gray (Gy)] GL261xRAG-neo hybrids (10^6^ cells per mouse) in 0.5 mL PBS, and control mice were injected s.c. with 0.5 mL PBS alone (mock-vaccination). Mice were checked daily until the end of the experiment. Mice were euthanized as soon as they looked sick. Length of animal survival was measured by means of Kaplan–Meier graphs. Data from the animal experiments were analyzed using GraphPad Prism software program (GraphPad Software Inc., La Jolla, CA, USA), with p < 0.05 indicating statistical significance. Approximately 120 mice were tested in these experiments, including mice used in preliminary studies aimed at optimizing the surgical procedures and determining the timing of vaccine administration. Less than 5 % of mice receiving intracranial inoculation of GL261 cells died within 48 h after surgery; these mice were excluded from the survival analyses.

### Bioluminescence Imaging (BLI)

For BLI studies, luciferase activity was monitored using an Ivis 200 Spectrum Instrument (Perkin Elmer, Waltham, MA). Two and four days post-implantation of GL261 cells in the brain, mice were anesthetized in a chamber by inhalation of isoflurane (Florane, Baxter) at 2–3 % in oxygen for the duration of the imaging session, and injected i.p. with 150 μL (150 mg/kg) of the bioluminescent substrate ReadyJect D-Luciferin (PerkinElmer). Up to three mice were placed within the BLI chamber and imaged until measurable luciferase activity could be recorded. Serial images were obtained at 1, 4, 7, and 10 min after i.p. injection of luciferin. Mice were imaged using the following parameters: field of view C, exposure time 60 s, medium binning and F/Stop:1. Luciferase activity was calculated using Living Image Software (Perkin Elmer).

### Pathology and immunohistochemistry

At time of sacrifice, mouse brains were removed and fixed for 24 h in 4 % paraformaldehyde in PBS. After fixation, brains were immersed in 30 % sucrose for cryosectioning; sections were 5–30 μm-thick depending on the study. For morphological analysis, sections were stained with Hematoxylin and Eosin (H&E). Tumor volumes (in mm^3^) were estimated using image analysis (NIH Image software ImageJ 1.37v).

For immunohistochemical studies, tissue sections were placed into wells containing 0.5 mL of Tris-buffered saline (TBS) and incubated in blocking buffer (TBS + 5 % FBS) for 1 h at room temperature. Primary antibodies diluted in blocking buffer were applied for 24 h at 4 ^°^C. Primary antibodies used were: microglia-specific anti-Iba1 (1:500; Wako cat.# 019-19741); anti-Ki-67 (1:100 abcam cat.# ab16667); anti-CD3 (1:100 abcam cat.# ab5690); anti-CD4 (1:100 abcam cat.# ab25475); anti-CD8 (1:100 abcam cat.# 22378). After the first incubation, sections were washed in TBS three times for 5 min and incubated for 60 min with secondary antibody (VECTSTAIN ABC Kit), followed by development using 3,3′-diaminobenzidine (DAB) horseradish peroxidase (HRP) substrate (Vector Laboratories). Control sections were processed in identical fashion, except that the primary antibodies were omitted.

## Results

**Fig. 1:**
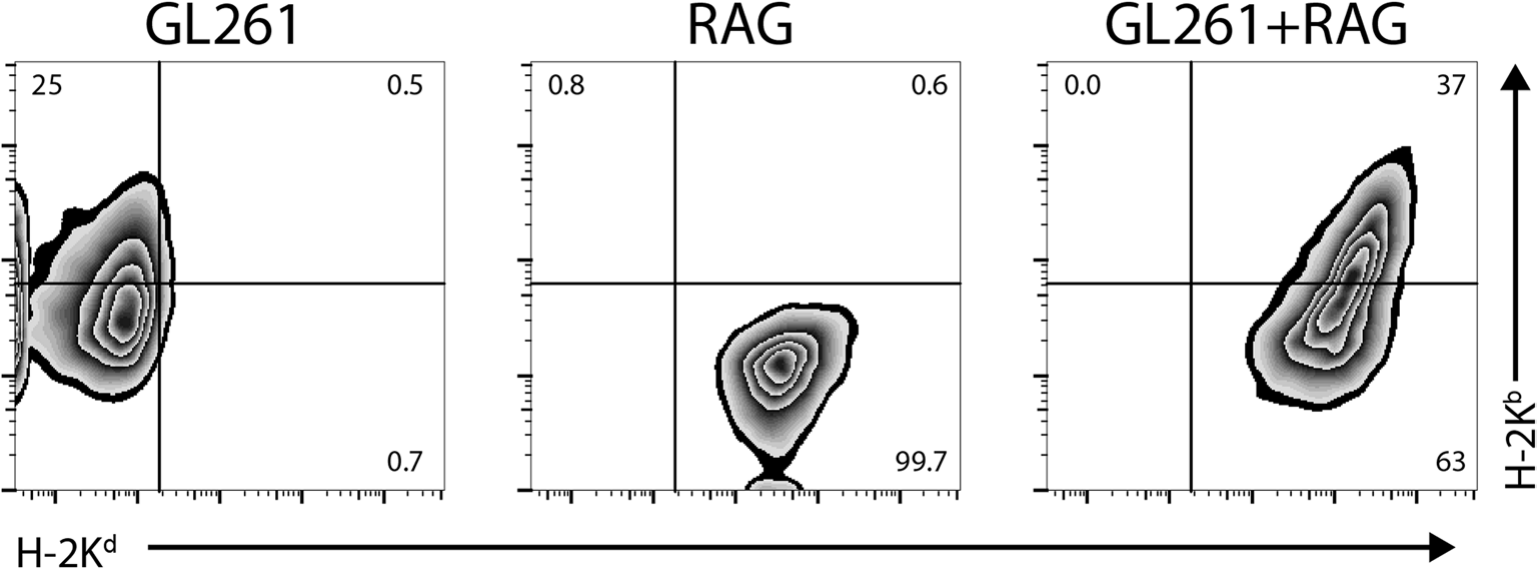
Cell surface staining of parental (GL261 and RAG) cells and semi-allogeneic somatic cell hybrids (GL261xRAG). Single-cell suspensions of each cell line were incubated with PE-labeled, H-2K^b^-specific MAb AF6-88.5, and APC-labeled, H-2K^d^-specific MAb SF1-1.1. Cell surface expressions of H-2K^b^ and H-2K^d^ antigens were measured by flow-cytometry. *Dot-plots* show that GL261 cells only express the H-2K^b^ antigen, RAG cells only express the H-2K^d^ antigen, while GL261xRAG cells express both antigens

Single-cell suspensions of GL261 and RAG-neo cultures were fused using PEG 1450 and semi-allogeneic somatic cell hybrids were selected in DMEM + 10 % FBS, containing 400 µg/mL G418 and HAT supplement. HAT-resistant and neomycin-resistant cell colonies became visible after 2–3 weeks; drug-resistant cultures were expanded and tested by FACS for expression of cell surface antigens derived from each parental cell. As expected, semi-allogeneic somatic cell hybrids expressed both H-2K^b^ (GL261) and H-2K^d^ (RAG-neo) MHC antigens (Fig. 1).

**Fig. 2:**
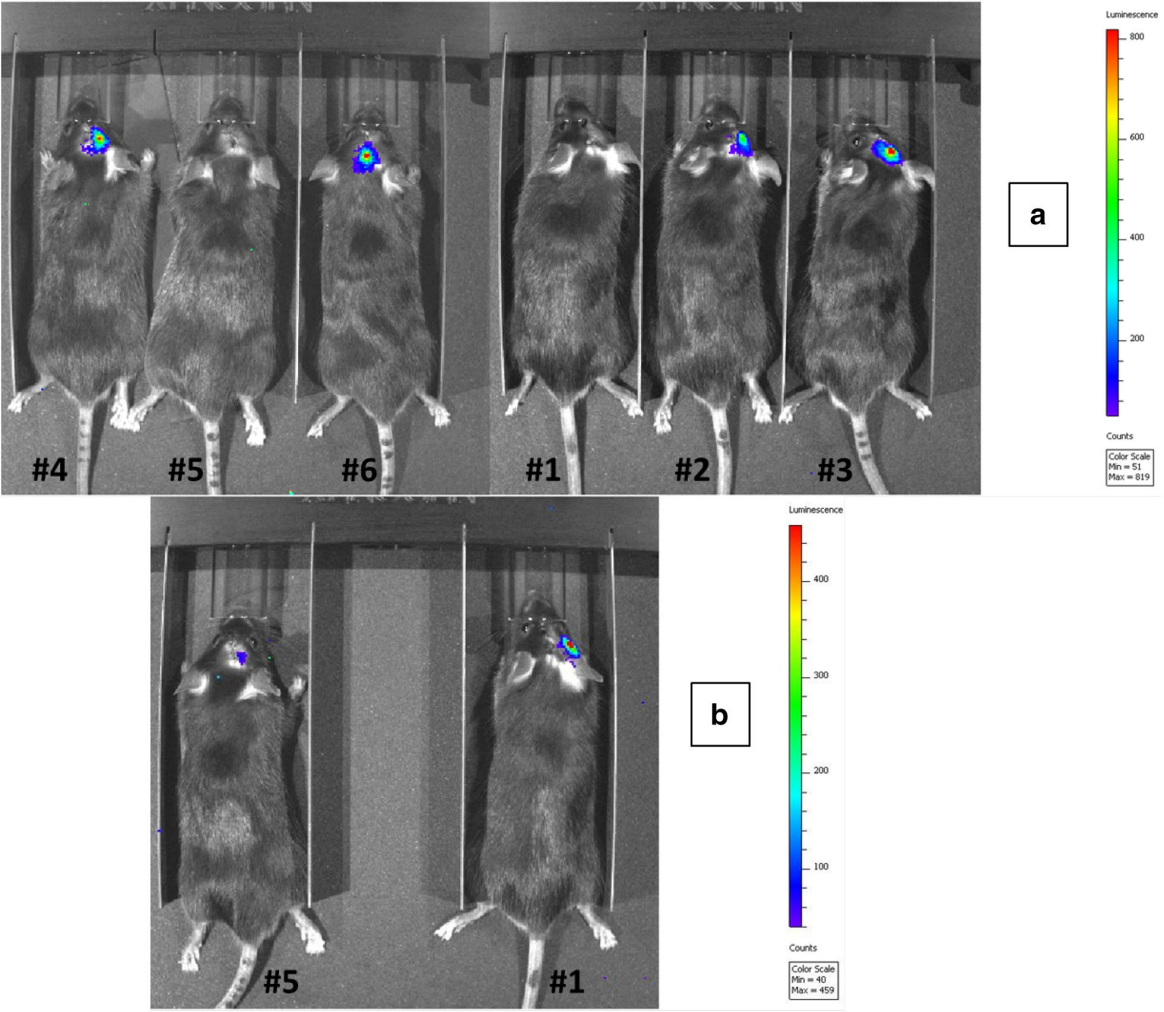
Bioluminescence Imaging (BLI). All mice received an intracranial injection of 2 × 10^4^ GL261 cells/mouse. For BLI, mice were anesthetized in a chamber containing a mixture of isoflurane (Florane, Baxter) and injected i.p. with 150 μL of the bioluminescent substrate ReadyJect d-Luciferin. Mice were placed within the chamber of a Xenogen IVIS 200 Bioluminescence and Fluorescence Imaging System for up to 10 min and sets of serial images obtained. Eight mice were imaged 2 days post-implantation of GL261 cells and six of them (75 %) showed measurable luciferase signal within the brain. **a** shows six of the eight imaged mice, including mice #1 and #5 which showed no signal. On day four post-implantation, both mice #1 and #5 showed a positive signal within the brain (**b**)

We wanted to establish how soon GL261-derived tumors were detectable after intracranial injection of 2 × 10^4^ GL261 cells per mouse. GL261 cells express a transfected gene coding for the firefly luciferase, which allows us to monitor tumor engraftment by bioluminescence imaging (BLI). Eight mice were imaged 2 days post-implantation of GL261 cells and six of them (75 %) showed measurable luciferase activity within the brain, demonstrating the presence of metabolically active tumor cells. Figure 2a shows six of the eight mice imaged 2 days post-implantation of GL261 cells, including mice #1 and #5 which appeared to be negative. On day four post-implantation, both of these mice showed luciferase activity within the right hemisphere of the brain, demonstrating that the implanted GL261 cells, although not visible 2 days after inoculation in these mice, became detectable later because they grew over time (Fig. 2b).

**Fig. 3:**
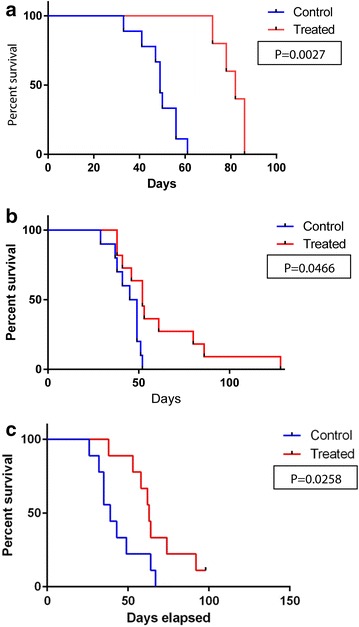
Survival* graphs*. **a**–**c** show Kaplan–Meier survival* graphs* of vaccinated (treated) vs. mock-vaccinated (control) mice. Three days after intracranial injection of 2 × 10^4^ GL261 cells, control mice were mock-vaccinated s.c. with 0.5 mL PBS (*blue graphs*), while treated mice were vaccinated with lethally irradiated GL261xRAG-neo hybrids (10^6^ cells/mouse) in 0.5 mL PBS (*red graphs*). Overall survival of vaccinated mice was significantly longer (p = 0.0027, p = 0.0466, and p = 0.0258, respectively)

**Fig. 4:**
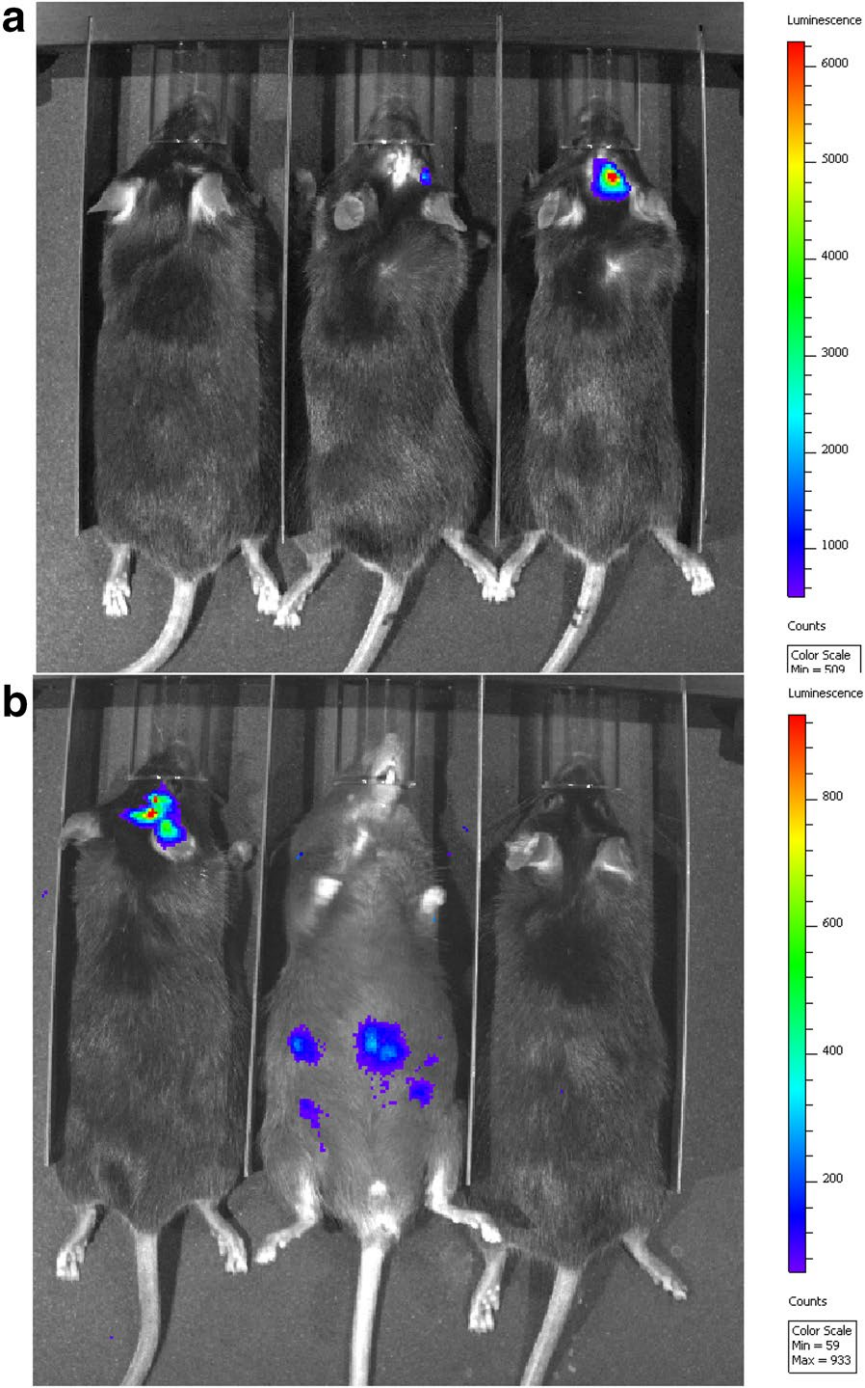
BLI of long-term surviving mice. The vaccinated mouse shown on the* left* in figure **a** underwent BLI at 99 days post-engraftment of GL261 cells; two control tumor-bearing mice are shown at the* center* and* right*. The vaccinated mouse shown on the* right* of figure **b** underwent BLI at 93 days post-engraftment with 2 × 10^4^ GL261 cells; two control tumor-bearing mice are shown at the* center* and* left*. Both vaccinated, long-term surviving mice appeared to be disease-free

In order to test GL261xRAG-neo hybrids as therapeutic vaccines against GL261-derived glioma, 10 week-old C57BL/6 mice were administered under anesthesia an intracranial injection of 2 × 10^4^ GL261 cells per mouse and after 3 days mice in the experimental group received a s.c. injection of irradiated GL261xRAG-neo hybrids (10^6^ cells per mouse) in PBS, and control mice were injected s.c. with PBS alone. We observed a significant longer survival in mice receiving the vaccine compared to control mice (Fig. 3a–c; p = 0.0027, p = 0.0466, and p = 0.0258, respectively). The longest-surviving vaccinated mice from experiments shown in Fig. 3b, c were imaged by BLI at 99 and 93 days post-implantation of GL261 cells, respectively, and compared to tumor-bearing mice as positive controls (Fig. 4). We observed no bioluminescence in the brain of the vaccinated mice compared to the control mice, which exhibited a strong positive signal, suggesting that the longest-surviving vaccinated mice were disease-free. The BLI-negative mouse shown in Fig. 4a had previously shown strong brain bioluminescence 2 days post-implantation of GL261 cells (mouse #2 in Fig. 2a) and was sacrificed 128 days post-implantation to collect spleen and cervical lymph nodes (no tumor was observed in the brain). The BLI-negative mouse shown in Fig. 4b was sacrificed at day 105 post-implantation to collect spleen and cervical lymph nodes. The results of these animal experiments are consistent with the estimate that 5-10 % of mice receiving a single vaccine administration became long-term survivors and appeared to be disease-free (and possibly cured) by BLI.

**Fig. 5:**
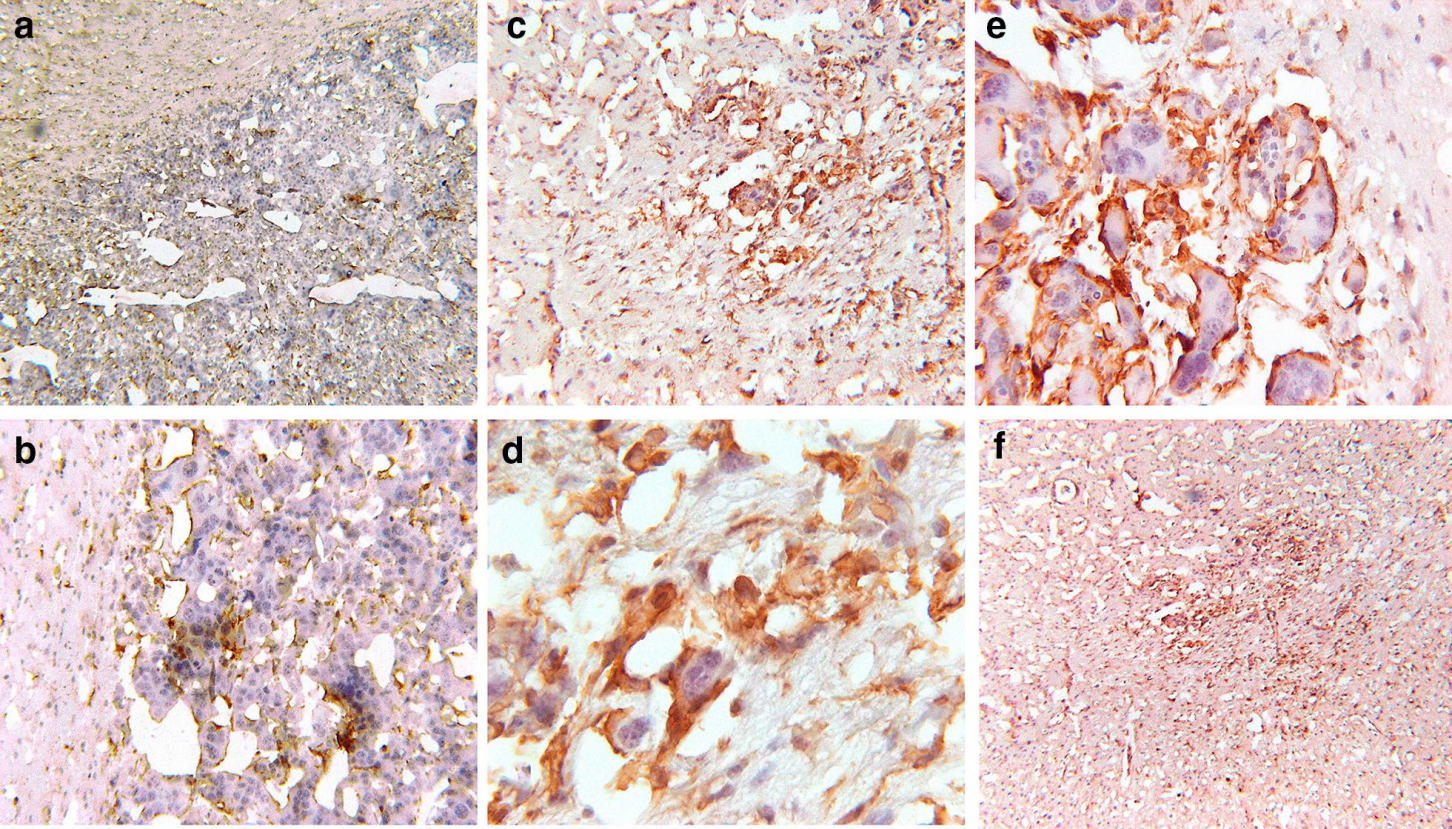
Immunoperoxidase staining of microglia in brain sections from mock-vaccinated and vaccinated mice. **a** The large tumor from a mock-vaccinated mouse occupies the central, lower and right areas in this image. Uninvolved brain is in the *upper left quadrant* of the image. *Brown staining marks* microglia in the surrounding brain and in the tumor. The border of the tumor with the surrounding brain is distinct but ragged (magnification ×20). **b** This photomicrograph from the same mock-vaccinated mouse shows the interface of tumor and adjacent brain. Staining for microglia is present in both. Tumor cells are hyperchromatic and atypical mitoses are present. A thin rim of microglia staining adjacent to tumor cells is visible (magnification ×100). **c** This photomicrograph shows a much smaller tumor in a vaccinated mouse. Microglia cells are intermingled within the tumor, attached to tumor cells, and are more numerous in surrounding brain. Microglia cells are also more numerous around tumor cells in vaccinated mice compared to mock-vaccinated ones (magnification ×40). **d** In this photomicrograph from the same vaccinated mouse, numerous microglia cells surround glioblastoma cells, distinguished by their nuclear atypia, pleomorphism, nucleomegaly and nucleoli. Microglia have much smaller nuclei and their cytoplasm is marked by the immunoperoxidase stain (magnification ×200). **e** In a different vaccinated mouse, microglia surround tumor cells, which are very large and have nucleomegaly with multiple nucleoli. Stained microglia cells are located on the perimeter of the malignant cells (magnification ×400). **f** This low-power image of a small tumor from a vaccinated mouse shows the concentration of staining within the tumor and the large number of activated microglia in the surrounding neuropil (magnification ×20)

Brain tissue from mock-vaccinated mice (PBS alone) and two vaccinated mice sacrificed 86 days post-implantation of GL261 cells (from survival experiment shown in Fig. 3a) were processed for histological examination and sections prepared for immunohistochemical staining, using as primary antibody a rabbit polyclonal antiserum specific for “ionized calcium binding adaptor molecule 1” (Iba1), a well-known marker for activated microglia (Fig. 5). We observed that microglia cells were less numerous within and around the tumor of mock-vaccinated mice (Fig. 5a, b), compared to vaccinated mice (Fig. 5c–f). Activated microglia appeared to form a fence along the perimeter of the tumor cells (Fig. 5e). We also used antibodies specific for CD3, CD4, and CD8 and observed lymphocytic infiltrates; in contrast to what observed with microglia, there were no obvious differences in tumor sections from vaccinated mice compared to tumor sections from mock-vaccinated mice (data not shown). Brain sections were also stained with anti-Ki67 rabbit monoclonal SP6 (abcam, Cambridge, MA, USA) to investigate whether there were differences in the growth fraction of tumors from vaccinated mice compared to tumors from mock-vaccinated mice. We observed that the intensity and prevalence of Ki67-positive cells was comparable between the two groups and that in large tumors Ki67-positive cells were especially numerous at the edge of the tumor (data not shown).

**Fig. 6:**
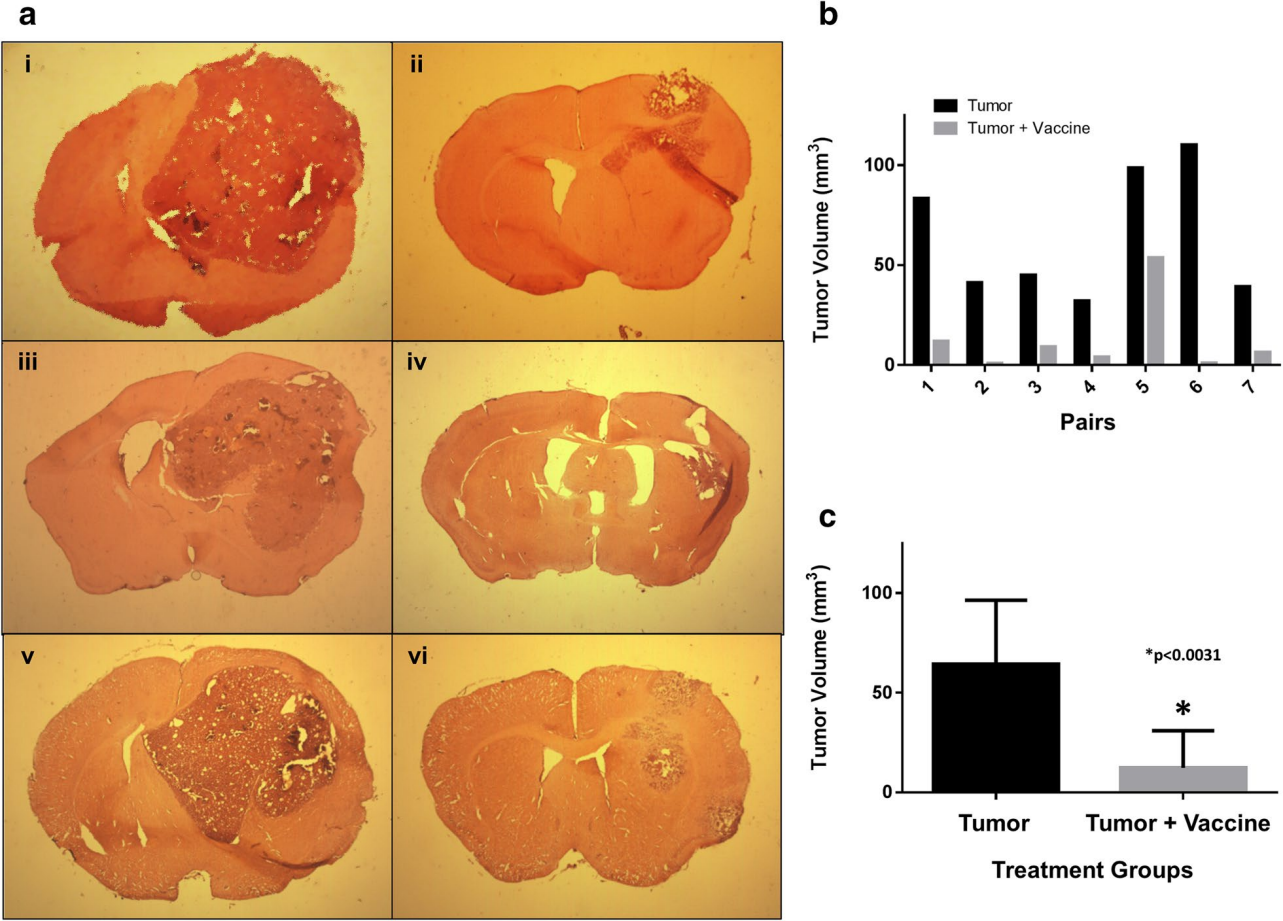
Comparison of tumor size in brain sections from vaccinated and mock-vaccinated mice. Animals from the two groups were sacrificed in pairs when any control mouse showed signs of discomfort. **a** shows corresponding sections of the brain (stained by H&E) from three pairs of mice for comparison. Tumors from mock-vaccinated mice (*i*, *iii*, *v*) occupied most of the *right hemisphere*. In contrast, tumors from vaccinated mice (*ii*, *iv*, *vi*) were much smaller. **b** shows the *bar graph* of tumor volumes for each of the seven pairs of mice. **c** shows the *bar graph* of the cumulative data; tumor volumes were significantly smaller in vaccinated mice compared to mock-vaccinated mice (p < 0.0031). Tumor volumes (in mm^3^) were estimated using image analysis (NIH Image software ImageJ 1.37v)

Because of the significantly longer survival of vaccinated mice compared to mock-vaccinated ones, we attempted to quantify the difference in tumor volume between the two groups. Fourteen mice were inoculated with 2 × 10^4^ GL261 cells/mouse injected into the right hemisphere and after 3 days seven of them were injected s.c. with lethally irradiated (30 Gy) GL261xRAG-neo hybrids (10^6^ cells per mouse). As soon as any mock-vaccinated mouse showed signs of stress or discomfort, it was sacrificed together with a randomly picked mouse from the vaccinated group and the brains processed for comparison of each pair. Figure 6a shows low-magnification sections of the brain from the first three pairs and demonstrate the striking difference in tumor size between the two groups at each time point. Comparison of tumor volumes based on serial sections of the brain from seven pairs of vaccinated and mock-vaccinated mice are shown in Fig. 6b, c. Tumor volumes were significantly smaller in vaccinated mice compared to mock-vaccinated mice (p < 0.0031).

## Discussion

We report on the efficacy of a therapeutic cancer vaccine in mice inoculated with a lethal dose of GL261 glioma cells in the right hemisphere of the brain. Overall survival of vaccinated mice was significantly longer compared to mock-vaccinated mice. Tumor volume in the brain of vaccinated mice was on average five to ten-fold smaller compared to mock-vaccinated mice. The significant differences in tumor size and overall survival between vaccinated and mock-vaccinated mice resulted from a single s.c. injection of irradiated vaccine. It must be noted that subcutaneous vaccination with irradiated GL261 cells alone was partially effective in a preventive setting, but totally ineffective in a therapeutic setting, i.e., when administered on the day of intracranial tumor inoculation or 3 days thereafter [8]. Our results show that irradiated semi-allogeneic vaccines are effective in a therapeutic setting.

In vaccinated mice, conspicuous microglia infiltrates were observed in tumor tissue sections and activated microglia appeared to form a fence along the perimeter of the tumor cells. Although these observations suggest an anti-GBM role for microglia infiltrates in vaccinated mice, the balance of published evidence suggests that glioma-infiltrating macrophages favor tumor growth and infiltration into normal brain [10–12]. Although we did not observe clear differences in T cell infiltrates between vaccinated and mock-vaccinated mice, this does not imply that T cells do not play an important role in the anti-tumor response. Studies involving knock-out mice missing specific T-cell or macrophage cell lineages together with in vitro studies are presently being conducted to properly address this important issue. Additional immunohistochemical studies involving vaccinated and mock-vaccinated mice sacrificed at regular intervals will also be informative and help us understand the role of cell types responsible for the anti-tumor effects.

We reported on the use of semi-allogeneic vaccines as stimulators of HIV-envelope-specific cytotoxic T lymphocytes (CTL) and we proposed that semi-allogeneic cell hybrids functionally mimic antigen-presenting cells (APC) by concomitantly stimulating alloantigen-specific T helper cells via allogeneic MHC, and antigen-specific CTL precursors via antigen presentation through self-MHC [13]. We also proposed that the Th-1 cytokine response, induced through alloantigen-specific help, activates more efficiently antigen-specific CTL and that the cytokine-rich microenvironment of allograft rejection is crucial to attracting dendritic APC [5].Taken together, the results of these previous studies suggest that the semi-allogeneic platform for therapeutic cancer vaccines is essentially made of three components: (1) A “self” component, represented by the host-derived MHC haplotype (e.g., H-2^b^) expressed by the tumor cells; (2) an “allo” (non-self) component represented by a different cell line (e.g., RAG), that has a different MHC (H-2^d^) haplotype; and (3) an “antigenic” component which is specific for the tumor (e.g., GL261). It should be possible to extend this immunotherapy approach to human subjects with GBM and test it through properly designed clinical studies. By fusing RAG-like human cells [9, 14] with single cell suspensions from each patient-derived GBM, the antigenic complexity and specificity of each tumor would be captured, resulting in a therapeutic vaccine that is representative of each patient’s GBM. Thus, the long-term objective of this translational research effort is to test irradiated, semi-allogeneic vaccines as an adjuvant to standard of care for human subjects diagnosed with GBM.

## Conclusions

The results of these animal studies persuaded the Office of Orphan Products Development of the Food and Drug Administration to grant Orphan Drug Designation to the corresponding author for treatment of GBM with irradiated, semi-allogeneic vaccines. Furthermore, these results are consistent with the recognition that immunotherapy has emerged as an important adjuvant in the therapeutic armamentarium of clinicians against GBM [15], and support the goal of translating our immunotherapy approach into clinical studies, with a special focus on Veterans with GBM, because of their significantly shorter survival compared to non-VA patients with the same diagnosis [3].

## Abbreviations

ATCC: American Type Culture Collection
BLI: bioluminescence imaging
CTL: cytotoxic T lymphocytes
DAB: 3,3′-diaminobenzidine
DMEM: Dulbecco’s modified Eagle’s medium
FACS: fluorescence-activated cell sorting
FBS: fetal bovine serum
FDA: food and drug administration
G418: geneticin
GBM: glioblastoma multiforme
Gy: gray
H&E: hematoxylin and eosin
HAT: hypoxanthine aminopterin thymidine
HGPRT: hypoxanthine–guanine phosphoribosyl transferase
HRP: horse radish peroxidase
Mab: monoclonal antibody
MHC: major histocompatibility complex
IFN: interferon
MUSC: Medical University of South Carolina
PEG: polyethylene glycol
PBS: phosphate-buffered saline
PD-1: programmed cell death protein 1
SDS: sodium dodecyl sulfate
TBS: tris-buffered saline
VA: Veterans Administration
